# One year of smokefree bars and restaurants in New Zealand: Impacts and responses

**DOI:** 10.1186/1471-2458-6-64

**Published:** 2006-03-14

**Authors:** George Thomson, Nick Wilson

**Affiliations:** 1 Department of Public Health, Wellington School of Medicine and Health Sciences, University of Otago, Box 7343 Wellington South, New Zealand

## Abstract

**Background:**

New Zealand introduced a smokefree bars and restaurants policy in December 2004. We reviewed the data available at December 2005 on the main public health, societal and political impacts and responses within New Zealand to the new law.

**Methods:**

Data were collected from publicly available survey reports, and from government departments and interviews. This included data on smoking in bars, attitudes to smokefree bars, bar patronage, socially cued smoking, and perceived rights to smokefree workplaces.

**Results:**

The proportion of surveyed bars with smoking occurring decreased from 95% to 3% during July 2004 – April 2005. Between 2004 and 2005, public support for smokefree bars rose from 56% to 69%. In the same period, support for the rights of bar workers to have smokefree workplaces rose from 81% to 91%. During the first ten months of the smokefree bars policy, there were only 196 complaints to officials about smoking in the over 9900 licensed premises. The proportion of smokers who reported that they smoked more than normal at bars, nightclubs, casinos and cafés halved between 2004 and 2005 (from 58% to 29%).

Seasonally adjusted sales in bars and clubs changed little (0.6% increase) between the first three quarters of 2004 and of 2005, while café and restaurant sales increased by 9.3% in the same period. Both changes continued existing trends. Compared to the same period in 2004, average employment during the first three quarters of 2005 was up 24% for 'pubs, taverns and bars', up 9% for cafés/restaurants, and down 8% for clubs (though employment in 'pubs, taverns and bars' may have been affected by unusually high patronage around a major sports-series).

The proportion of bar managers who approved of smokefree bars increased from 44% to 60% between November 2004 and May 2005. Bar managers also reported increased agreement with the rights of bar workers and patrons to smokefree environments. The main reported concerns of the national and regional Hospitality Associations, in 2005, were the perceived negative effects on rural and traditional pubs.

**Conclusion:**

As in other jurisdictions, the introduction of smokefree bars in New Zealand has had positive overall health protection, economic and social effects; in contrast to the predictions of opponents.

## Background

In December 2004, the New Zealand *Smokefree Environments Amendment Act *of 2003 was implemented. This Act had the effect of making nearly all workplaces and associated facilities (eg, warehouses, factories and workplace lunchrooms) smokefree. The policy includes all places licensed to sell alcohol or food, and is for all areas in such premises that are 'substantially enclosed'. A media campaign on not smoking in workplaces was run during August-December 2003, one on not smoking in homes was run from April 2004, and one on the new smokefree legislation was run in two parts in late 2004 and in early 2005 [1-3]. The 2003 campaign featured the exposure of bar workers to tobacco smoke, and the early 2005 television advertisement showed a smokefree bar interior, with smokers outside. This article focuses on the effects of the 2003 Act's introduction of smokefree bars and restaurants.

Exposure to secondhand smoke (SHS) in workplaces has been declining in New Zealand, with 49% of adults being exposed at work in 1989 [4 pp.[49,50]]. Most offices and public interiors (eg, shops), and half of the seating in restaurants in New Zealand became smokefree after the 1990 *Smoke-free Environments Act*, with only 39% of adults being exposed at work in 1991 [5 pp.[58,60]] and 34% in 2001 [6 p.[8]].

Before the new legislation came into effect in December 2004, SHS was estimated to cause over 300 deaths a year in New Zealand, with 100 deaths resulting from workplace exposure to SHS [7]. There are over 9900 places in New Zealand licensed for selling alcohol on the premises in 2005 (Pers. comm. B Holmes of Liquor Licensing Authority to G Thomson, 13 December 2005) with over 65,000 employees in bars, clubs, cafés and restaurants (some are in non-licensed premises) [8]. International studies have shown that bar and restaurant workers are an occupational group that is highly exposed to SHS [9,10].

Similarly, prior to the 2003 Act, New Zealand studies found that non-smoking bar workers were exposed to levels of SHS at work that were higher than the levels away from work, and higher than the levels for workers in smokefree workplaces. This was demonstrated by increased cotinine levels during their work shifts, and by nicotine levels in the hair of a range of workers [11-13]. A survey in one city in 1999–2000 found that 77% of workers in licensed premises were exposed to SHS, and over half of those exposed reported irritation from SHS to their throat or lungs [14].

Smoking in bars can also be a major source of smoking normalisation, a source of cues for smoking [15] (which may increase consumption and undermine quit attempts), and may increase the risk of youth smoking uptake [16]. Smokefree workplace policies elsewhere have been shown to reduce such cues, decrease tobacco consumption, increase quit rates, and reduce health risks [17-22].

Given the importance of smokefree policies for bars for advancing tobacco control, we reviewed the data available at December 2005 on the main public health, societal and political impacts and responses within New Zealand to the new law on smokefree bars and restaurants. Overall, we found positive responses and effects.

## Methods

Data sources for this review were national surveys conducted by the National Research Bureau, Health Sponsorship Council, TNS New Zealand and Gravitas. These surveys were on smoking in bars, attitudes to smokefree bars, bar patronage, socially cued smoking, and rights to smokefree workplaces [23-26]. Other data were obtained from the Liquor Licensing Authority (number of licences), New Zealand Customs (for tobacco released from bond), Statistics New Zealand (for retail sales and employment), and by interviews with government officials. Additional information was found in two reports on the impacts of the new law [27,28]. Searches for data in secondary literature were conducted in Medline, Index New Zealand and Google Scholar.

The Factiva database of print media was searched for all items (articles, editorials, letters) in 12 New Zealand newspapers (11 dailies and 1 Sunday paper) from January 2000 to 31 December 2005. These included six provincial area newspapers. We searched for items containing the words 'bars and (smoking or smokers or smoker or smoke),' which were about tobacco smoke in licensed premises. Monthly and yearly totals of items were made, and the items searched for (i) comments by representatives of the hospitality industry, and (ii) editorials.

We did not use the available data on the number of liquor licenses, due to quality of the Liquor Licensing Authority database, where there is only an occasional process of editing and rationalising (Pers. comm. B Holmes of Liquor Licensing Authority to G Thomson, 13 December 2005).

Cafés/restaurants are in New Zealand generally distinguished from pubs, taverns and bars by having the sale of food as their principal business.

## Results

### Expansion of smokefree areas

The new law resulted in a change from smoking being normal inside nearly all bars, to occurring in very few. An April 2005 survey of 193 bars by the National Research Bureau found smoking in only five (3%), compared to 183 (95%) in July 2004 [23]. Only 0.23% of the patrons present at the time of the April 2005 survey were smoking, compared to 24% of the patrons in July 2004. Most of the smoking found in the 2005 survey was in only one bar, where six of 18 people were smoking.

The policy change and the surrounding publicity may also have contributed to the increased prevalence of smokefree homes. Whereas the proportion of people that reported not being exposed to SHS at home in the last seven days increased by a non-significant amount between 2003 and 2004 (80.1% to 81.1%), between March 2004 and March 2005 the prevalence increased by 4% from 81.1% to 85.1% (95%CI for the 85.1% result: 83.7–86.5) [25].

### Impact on quitting, uptake and consumption

One study of calls to the national Quitline found statistically significant short-term changes associated with the introduction of smokefree bars. There were increases in calls, and in the dispensing of nicotine replacement therapy vouchers, during the month when, and month after, the smokefree bars law came into force in December 2004, compared to the same two months the year before [29]. However, the call levels since this time have not been systematically analysed to adjust for varying levels of advertising of the Quitline service.

Tobacco sales in supermarkets and service stations declined by 1.5% during the year to October 2005 compared to the year to October 2004 [30].

Making public social venues (bars, nightclubs, casinos and cafés) smokefree appears to have had an effect on smoking *levels *reported by smokers at these settings. The proportion of smokers who reported that they smoked "more than normal" when at such venues halved between 2004 and 2005 (57.8% to 28.6% [95% CI: 24.3–32.9]). However, 76% of smokers reported that they still smoked (if generally less) when attending these venues, presumably in the outdoor areas [[25] Table 11].

### Support for smokefree bars and restaurants

Public support can be measured in several ways, including responses to questions about support for making particular venues smokefree, by attitudes to rights of workers to smokefree workplaces, and by reported patronage of bars and cafés/restaurants. The latter is reported on in the subsequent section on economic impacts.

**Figure 1:**
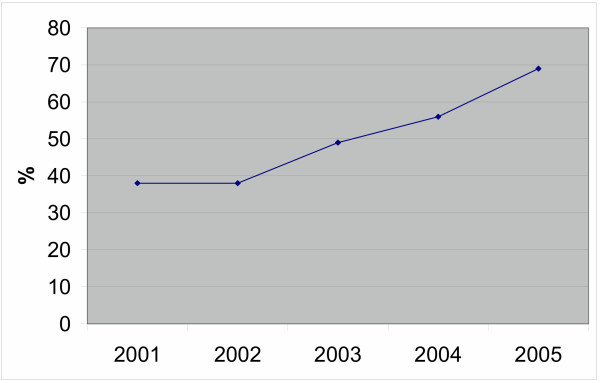
**Public support for completely smokefree pubs and bars**. National survey data from UMR Research Ltd [26].

Serial national surveys (n = 750 respondents) show that support for smokefree policies for 'pubs and bars' almost doubled between 2001 and 2005 (Figure 1). Support increased from 56% to 69% between November 2004 and April 2005 (after implementation of the Act), with support from smokers increasing from 22% to 42% in that period [26].

Public support for the rights of workers to smokefree workplaces, in both bars and cafés/restaurants, also increased significantly since 2001, and continued to increase between 2004 and 2005 (80.6% [95% CI: 79.0–82.2] to 90.8% [95% CI: 89.6–92.0] for bar workers; and 85.8% [95% CI: 84.4–87.2] to 93.7% [95% CI: 92.7–94.7] for café and restaurant workers) [[25] Table 8; 31 p.[67]].

### Economic impact on the hospitality sector

**Figure 2:**
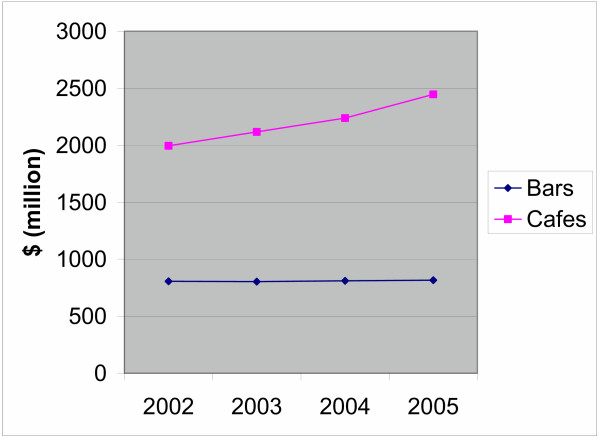
**National sales volumes for bars and clubs compared to cafés and restaurants: January-September for 2002–2005**. National data from Statistics New Zealand [32].

Retail sales data over the last four years indicate that the bar and club sector has had stagnant growth, compared with the café and restaurant sector (Figure 2). Seasonally adjusted sales at bars and clubs, during the periods January-September for the years 2002–2005, show that both before and after the smokefree policy change, the bar and club sector was static. Compared to the same period the year before, sales in 2003 were 0.25% down, in 2004 they were 0.9% up, and in 2005 they were 0.6% up. This is in contrast to the growth in café and restaurant sales, of 6.0%, 5.7% and 9.3% for the years 2003–2005 respectively, and in the whole retail sector, of 5.3%, 7.6% and 7.1% for the years 2003–2005 respectively [32].

**Figure 3:**
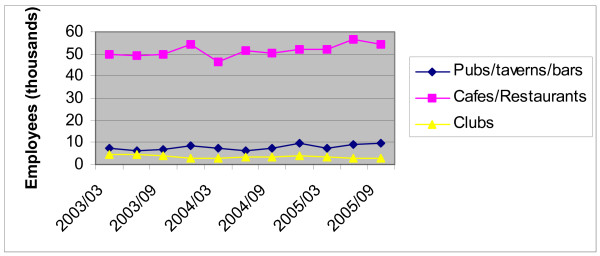
**Employment in licensed premises, restaurants and cafés 2003–2005 (in full-time equivalents)**. National data from Statistics New Zealand [8].

During the three years 2003–2005, employment in licensed premises, cafés and restaurants has been static or slightly increasing (Figure 3) [8]. Compared to the same period in 2004, average employment (full-time equivalents) during the first three quarters in 2005 was up 24% for 'pubs, taverns and bars', up 9% for cafés and restaurants, and down 8% for clubs. Interpretation of these data is complicated by the 2005 period including the British Lions rugby tour of New Zealand, which may have increased hospitality sector spending by fans.

Surveys of the public indicate that while reported bar visits (at least monthly) by smokers remained static, bar visits by non-smokers increased markedly between 2004 and 2005, from 35.4% up to 49.4% (95% CI: 47.1–51.7). Both reported smoker and non-smoker visits to cafés increased from 2004 to 2005 (smokers' from 65.8% to 69.2% [95% CI: 65.6–72.8], non-smokers' from 65.5% to 73.4% [95% CI: 71.4–75.4]) [[25] Table 10].

### Attitudes of key interest groups

Repeat surveys were conducted of a cohort of 346 bar managers (44% were also bar owners) before and after the introduction of smokefree bars (November 2004 and May 2005). These indicate that support for smokefree bars increased from 44% to 60% after the smokefree legislation came into force (an absolute increase of 16%, 95% CI: 10–23%) [24]. Only 18% disapproved of smokefree bars in 2005. The proportion of managers agreeing that bar workers have a right to work in a smokefree environment increased from 55% to 62% (an absolute increase of 7%, 95% CI: 0–14%), with only 15% disagreeing. In 2004, 53% of bar managers agreed that bar *patrons *had a right to a smokefree environment, with the proportion increasing to 65% in 2005 (an absolute increase of 12%, 95% CI: 4–20%).

In the period 2000–2004 there were consistent survey and newspaper reports of the fears of publicans about decreased revenue after the introduction of the smokefree policy, and problems of implementing the proposed policy [33,34]. The Hospitality Association of New Zealand (HANZ) opposed the idea of smokefree bar laws for a number of years, predicting significant losses in jobs and incomes, and business closures. The HANZ chief executive in 2004 claimed large drops in bar revenue in Ireland and New York as the result of smokefree policies [35].

Since the introduction of the smokefree legislation, the response from hospitality organisations and spokespeople has been mixed, as the quotations below suggest. These quotations are representative of the coverage of the spokespeople found. In July 2005, the HANZ chief executive was reported as saying that 'both rural and suburban pubs had already suffered a decline in patrons after the ban was introduced' [36]. In October 2005 he was reported as saying that proposed changes that would reverse the protection from SHS for some bar workers 'could help the industry as long as they were applied evenly to all licensed establishments' [37]. In December 2005 he was reported as saying that the overall maintenance of the number of smokers going to pubs was a 'testament to the industry's hard work in educating smokers' and that there were difficulties for some rural pubs, those with a 'traditional clientele' and those without an outdoor area [38].

In December 2004, the president of the Canterbury branch of HANZ was reported as saying 'We're getting some positive feedback, especially from the non-smokers. A lot more diners are also coming in because it's not so smoky' [39]. After the introduction of the smokefree bars policy, the Nelson district HANZ president was reported as saying that there were few enforcement problems [40]. A year later, he commented on the difficulties for 'the more traditional blue-collar hotels' [40]. At the same time, the president of the Bay of Plenty branch of HANZ commented that 'the traditional Kiwi pubs patronised by "blue collar" drinkers had been hit hard' [41].

### Cost of enforcement

In the first weekend of smokefree bars and restaurants in December 2004, only 50 calls to the free Ministry of Health phoneline were made nationally. Most were from bar owners and managers asking for information, with only 12 calls reporting someone smoking in a bar [42]. After four months there had been 94 complaints about smoking in licensed premises [43], and after ten months, 196 complaints [28].

By December 2005, legal action had been taken against four publicans for allegedly breaching the smokefree law. One was convicted and fined $NZ9000 [44], one had terminated the legal action by filing for bankruptcy [45,46], and two actions were ongoing. In one of the latter cases, the local Liquor Licensing Authority removed the licence for three weeks as a penalty for activity that included allowing smoking indoors [47,48].

The police are not directly involved in the enforcement of this smokefree law, unless they are called in because of violent behaviour or the illegal sales of alcohol. There have been some reports of an increase in unlicensed bars in private premises (eg, in home garages), but no substantiated numbers [36,49-51]. These bars are illegal if alcohol is sold.

### The political response

When the law was passed, it was supported by three of the seven political parties in the Parliament, along with a few Members of Parliament (MPs) from three other parties [52]. Opposition politicians predicted decreased hospitality sector employment [53], and that 'many' bars would go out of business [54]. In mid-2005 nearly all the MPs from four opposition parties voted for a private member's Bill introduced by an opposition MP to remove some workplace SHS protections (eg, for clubs) [55]. However, this Bill was defeated on its first reading.

The main opposition party, National, has largely opposed the smokefree bars legislation. In January 2005, the party leader (Don Brash) called for referenda on 'major constitutional and moral issues', including the smokefree bars policy [56]. After the October 2005 election, Dr Brash appointed an official 'Political Correctness Eradication' spokesperson for his party. The 'eradicator', MP Dr Wayne Mapp, targeted the smokefree bars legislation, and was reported as describing the law as 'nanny state' [37].

A new political party (WIN) was formed in March 2005 specifically to contest the smokefree bars policy. However, it withdrew from the election process in August 2005, and asked supporters to vote for a small party already in Parliament (United Future) [57,58]. The WIN party leader became a United Future party candidate [59] and won 2% of the votes in the electorate he stood for [60]. The United Future party lost five of its eight seats in Parliament in the October 2005 election (winning 2.7% of all votes), but had already been at below 3% in polls during most of 2005 [61].

### Newspaper coverage of SHS issues in bars

News coverage about smoking in bars in the 12 selected newspapers peaked in December 2004 (when the new legislation was implemented). The coverage then remained at the level of 9–27 per month during 2005 (ie, the year it was implemented).

**Figure 4:**
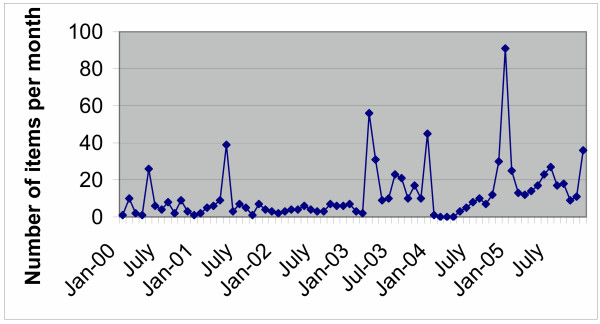
Newspaper items during 2000–2005 on secondhand smoke and licensed premises, in 12 New Zealand newspapers*. *Sunday Star Times, Dominion Post, Dominion, Evening Post, New Zealand Herald, Otago Daily Times (from 20 August 2002 only), Christchurch Press, Nelson Mail, Southland Times, Timaru Herald, Waikato Times, [Taranaki] Daily Times). Two of these (Dominion, Evening Post) were replaced in 2002 by the Dominion Post.

There were 316 newspaper items on smoking in bars in the period 1 December 2004 – 31 December 2005. The yearly totals of items, from 2000 to 2005, were 73, 91, 55, 237, 167 and 222. The monthly peak of 91 items in December 2004 was in contrast to peaks of 56 in March 2003 (when the report of the Parliamentary Select Committee on the Bill indicated the total ban intention) and 45 in December 2003, when the legislation was passed by Parliament (Figure 4).

The pattern of editorials in the selected newspapers varied over the period from May 2001 to December 2005. For the peak months in that period for numbers of newspaper items about smoking in bars (May 2001, March 2003, December 2003/2004/2005), there were 25 editorials in nine papers (three metropolitan, six provincial). Thirteen were generally positive, two had mixed views on the policy change, and ten were generally negative. There were five in May 2001 (one negative, four positive), eight in March 2003 (five negative, three positive), three in December 2003 (two negative, one positive), seven in December 2004 (one negative, two mixed, four positive), and two in December 2005 (both positive).

Analysis by geographic area suggested marked patterns. All but one of the 12 editorials in South Island papers were positive, compared to the nine negative, two mixed and two positive editorials in North Island papers. In the first three months of high newspaper attention to the issue of smoking in bars (May 2001, March and December 2003), all the eight editorials in North Island papers were negative.

In the five 'high attention' months, there were eleven editorials in the three highest circulation papers. The *Dominion Post *produced four editorials (all negative), the Christchurch *Press *three positive editorials, and the largest circulation paper, the *New Zealand Herald *one negative, one mixed and two positive. The only negative editorial from the *New Zealand Herald *was in March 2003, with the positive editorials in December 2004 and 2005.

### Other possible consequences

A downturn in the revenue from gambling machines in New Zealand casinos, during January-June 2005, is suggested by a government report. The report also indicates that spending on non-casino gambling machines has declined against a long-term trend. However, the gambling spending downturn may have been due to a range of factors, including the implementation of new gambling legislation that limited gambling machine numbers [62,63].

## Discussion

### Main findings

As in other jurisdictions with smokefree bars and restaurants [20,21,64-67], the policy has greatly increased the protection of bar and restaurant workers and patrons from exposure to SHS. The trend of increased public support for smokefree bars and restaurants continued after the policy change, as did public support for the rights of hospitality workers to smokefree workplaces. Smokers reported smoking less than their normal amount while at licensed premises.

The available data indicates that the smokfree legislation has had little impact on sales and employment in the hospitality sector. Bar managers appear to have been favourably impressed by the reality of smokefree bars, and their attitudes to the rights of bar workers and patrons to smokefree environments have become more positive. There were no data on any transfers of consumer spending (from gambling or bars etc) to other sectors of the economy that involve discretionary spending.

The level of complaints indicates that there were problems with compliance with the new policy in less than two per cent of licensed premises. Less than one in every two thousand licensees had continued their non-compliance to the point of this triggering legal action by authorities.

While opposition political parties continued to try to modify the smokefree policy for all bars, these efforts have not achieved any success to date, despite the opportunity of the October 2005 general election and a single reading of a private member's Bill. Given the increasing public support, it appears unlikely that the opposition parties will continue to pursue the issue in a substantive way.

The survey of newspaper items indicates that the implementation period (December 2004–December 2005) had an even greater rate of print media interest than the thirteen months up to the passing of the legislation in December 2003. As elsewhere, the New Zealand experience indicates that heavy media coverage is likely to occur when smokefree bar legislation is considered and implemented. To help ensure that such coverage is well informed, the health sectors of jurisdictions proposing and introducing such policies could work to provide local media with the best available international evidence on the consequences of such changes (eg. [68]).

It is possible that gambling machine revenue in bars may have been (at least temporarily) affected by the smokefree legislation. Gambling on these machines may be reduced because of the need for gamblers to go outside to smoke, and thus break the 'trance-inducing rituals associated with gambling' [69,70]. However, such reductions may be for only a matter of months [69,70]. Two studies in the USA (of charitable gambling and of racetrack 'lottery terminals') reported no effects on gambling profits from smokefree policies [71,72].

### Limitations of the data and methods

Due to the lack of data, this review was not exhaustive on the impacts from, and responses to, the new law. For instance, data were not available on smoking prevalence after the Act's implementation, objective indicators of exposure and air quality in hospitality settings, on health impacts among hospitality sector workers, on how tourists perceived the law, or on such costs for hospitality proprietors as cleaning and insurance. No studies of direct health gains in New Zealand as a result of the smokefree bar law have yet been published, in contrast to other places [20,73]. However, such gains may be difficult to measure in the context of ongoing trends for declining cardiovascular disease rates and improvements in treatment. The most exposed group to SHS (bar and restaurant workers) have also not been studied in New Zealand (eg, in terms of asthma exacerbations and respiratory infections).

Tobacco consumption data: A workplace or public social space smokefree policy could be an influence on the amount of tobacco consumed, other factors being equal [18,19]. However, smokefree bars and restaurants, along with other new smokefree areas, will be only one factor in determining tobacco consumption. For instance, other factors that may have influenced the data on the level of consumption include the level of news media coverage of tobacco and health issues, the promotion of smoking cessation services (especially the Quitline), and the affordability of tobacco (which increased by 0.7% in 2005 compared to 2004 because of rising real incomes and dropping unemployment) [74].

As data on the tobacco sales in other types of convenience stores (besides service stations) and in licensed premises are not yet publicly available, the decline in sales in supermarkets and service stations is only part of the necessary information on consumption patterns. Tobacco released into the domestic market is not an accurate indicator of consumption for quarterly or annual periods, due to the large fluctuations in the amounts released by tobacco manufacturers. Over the period 2002–2005 there has been a trend of declining quarterly releases into the market [[27,28]; p.7], but several further years of such data may be necessary before any effect on the trend from the 2004 smokefree workplaces changes could be determined.

### Implications for further research

Because of the need for data from longer periods after the policy change, further research is desirable. Priority areas where additional research would be useful include: further attitudinal data from surveys (to assess trends in smoking denormalisation), changes in the exposure levels and/or health of bar and restaurant workers (relative to pre-2005 data or to other developed countries), total tobacco sales over several years before and after the change, and any health or other social changes due to reduced gambling in casinos and bars.

### Implications for policy

Making bars and restaurants smokefree provides a major opportunity for governments to not only protect workers and patrons, but also to help denormalise smoking and increase public awareness of the advantages of smokefree indoor areas. An overall strategy could use media campaigns, tobacco price changes, large increases in smoking cessation support capacity, or other means to significantly increase quitting, reduce tobacco consumption and smoking uptake, and to increase the prevalence of smokefree homes and cars.

Shortcomings in the law and its implementation: Because the New Zealand law does not include provisions about smoking within proscribed distances of doorways and windows, there is still some SHS exposure of bar and restaurant workers and patrons *inside*, due to those smoking outside near doorways and windows. Those sharing, or working in outdoor terraces and other areas with smokers, are also still exposed to SHS. Another limitation with implementation of this law was that it was not integrated with any tobacco price increases, or any major mass media campaigns to promote quitting.

## Conclusion

The new law has increased the protection of workers and patrons from SHS. Survey data indicates that the law is acceptable to a majority of citizens and to most bar managers. There is also some evidence that it has contributed to quitting behaviour in the short term, and to the further denormalisation of smoking. The fears of the Hospitality Association and some opposition politicians appear to have been largely unfounded.

As elsewhere, the introduction of smokefree bars and restaurants in New Zealand demonstrates that governments can expect positive overall health protection, economic and social effects. Such flagship policies provide opportunities for significant advances in tobacco control.

## Abbreviations

HANZ – Hospitality Association of New Zealand

MP – Member of Parliament

SHS – Secondhand smoke

## Competing interests

The authors have undertaken contract work for a range of organisations involved in tobacco control including: ASH NZ, NZ Cancer Society, NZ Heart Foundation, NZ Smokefree Coalition, the Quit Group and the Ministry of Health.

## Authors' contributions

NW conceived the project and both authors developed it. Both authors gathered and analysed data, and wrote the text.

## Pre-publication history

The pre-publication history for this paper can be accessed here:
